# Intervertebral Disc-on-a-Chip as Advanced *In Vitro* Model for Mechanobiology Research and Drug Testing: A Review and Perspective

**DOI:** 10.3389/fbioe.2021.826867

**Published:** 2022-01-28

**Authors:** Andrea Mainardi, Elena Cambria, Paola Occhetta, Ivan Martin, Andrea Barbero, Stefan Schären, Arne Mehrkens, Olga Krupkova

**Affiliations:** ^1^ Department of Electronics, Information and Bioengineering, Politecnico di Milano, Milan, Italy; ^2^ Department of Biomedicine, University Hospital Basel, University of Basel, Basel, Switzerland; ^3^ Department of Biomedical Engineering, University of Basel, Allschwil, Switzerland; ^4^ Department of Biological Engineering, Massachusetts Institute of Technology, Cambridge, MA, United States; ^5^ Spine Surgery, University Hospital Basel, Basel, Switzerland; ^6^ Lepage Research Institute, University of Prešov, Prešov, Slovakia

**Keywords:** intervertebral disc, mechanical loading, microphysiological device design, organ-on-a-chip, mechanobiology, degenerative disc disease (DDD)

## Abstract

Discogenic back pain is one of the most diffused musculoskeletal pathologies and a hurdle to a good quality of life for millions of people. Existing therapeutic options are exclusively directed at reducing symptoms, not at targeting the underlying, still poorly understood, degenerative processes. Common intervertebral disc (IVD) disease models still do not fully replicate the course of degenerative IVD disease. Advanced disease models that incorporate mechanical loading are needed to investigate pathological causes and processes, as well as to identify therapeutic targets. Organs-on-chip (OoC) are microfluidic-based devices that aim at recapitulating tissue functions *in vitro* by introducing key features of the tissue microenvironment (e.g., 3D architecture, soluble signals and mechanical conditioning). In this review we analyze and depict existing OoC platforms used to investigate pathological alterations of IVD cells/tissues and discuss their benefits and limitations. Starting from the consideration that mechanobiology plays a pivotal role in both IVD homeostasis and degeneration, we then focus on OoC settings enabling to recapitulate physiological or aberrant mechanical loading, in conjunction with other relevant features (such as inflammation). Finally, we propose our view on design criteria for IVD-on-a-chip systems, offering a future perspective to model IVD mechanobiology.

## 1 Introduction

Low back pain (LBP) is a prevalent health problem, with 80% of people suffering from it at least once in their lifetime (Wieser et al., 2011; Vlaeyen et al., 2018). A major cause of LBP is degenerative disc disease (DDD), an age-related pathology of the intervertebral disc (IVD) (Zhang et al., 2009; Wuertz and Haglund, 2013). While there is also some genetic predisposition (Trefilova et al., 2021), DDD is clearly associated with mechanical risk factors such as spine misalignment (e.g. scoliosis) or excessive IVD loads due to obesity or occupational hazards (e.g. heavy lifting), supporting the relationship between aberrant mechanical loading and IVD degeneration (Adams and Roughley, 2006; Stokes et al., 2011; Macedo and Battié, 2019). Despite the proven mechanical nature of DDD, mediators aberrant mechanosensing and mechanotransduction are still poorly understood, thus are not therapeutically targeted. The absence of appropriate preclinical IVD models is one of the reasons hampering these advancements. A better understanding of the molecular mechanisms leading from hyperphysiological mechanical loading to IVD degeneration, inflammation, and nociception, might reveal more effective therapeutic targets.

Mechanobiological responses of the IVD have been traditionally studied using macroscale devices and bioreactors (Molladavoodi et al., 2020). These systems are designed to apply loading patterns with different degrees of complexity (e.g. tension, compression, shear or their combination) to two-dimensional (2D) or three-dimensional (3D) cell-based models, IVD tissue explants, as well as whole IVD organs (Neidlinger-Wilke et al., 2014; Gantenbein et al., 2015; Shan et al., 2017; Peroglio et al., 2018; Pfannkuche et al., 2020). Devices applying mechanical load 2D cell cultures (e.g. capable of stretching (Cambria et al., 2020a)) simulate an oversimplified human body architecture, as cells in 2D rely on adherence to a flat surface thus lacking the support of the surrounding extracellular matrix (ECM) characterizing native tissues. On the contrary, 3D cell-based models utilize biomaterials to mimic the tissue microenvironment (Krupkova et al., 2014; Cambria et al., 2020b). In 3D, IVD cells produce their own ECM and generate an environment responsive to mechanical loading. Macroscale loading bioreactors integrating 3D cell constructs thus provide more physiological conditions over 2D cell cultures and enable the investigation of the mechanobiological response of whole tissues (Peroglio et al., 2018). The most relevant bioreactors for preclinical drug development still remain those capable of dynamic loading of whole IVDs of either human or animal origin (i.e. IVD isolated from large animals as cow, dog or sheep) (Gawri et al., 2011; Gantenbein et al., 2015). However, it is important to consider that such *ex vivo* loading systems mostly do not allow for testing large sample numbers at once. Animal models might mimic better the disease complexity but present inherent differences in loading patterns, genetics, and even cellular composition (e.g. presence of notochordal cells) with respect to human counterparts. Table 1 summarizes the major advantages and disadvantages of available IVD preclinical models.

**TABLE 1:** Advantages and disadvantages of IVD models. OoC = organ-on-chip.

	Animal models	Classic *in vitro* models	*Ex-vivo* organ culture	Macroscale bioreactors	OoC devices
• Advantages	• Most accurate recapitulation of the whole IVD	• Operational ease• Low cost• High throughput• repeatability	• Whole IVD recapitulation• Clinically relevant size	• 3D environments• Complex physicochemical stimulation• Whole IVD stimulation• Clinically relevant size	• Mimicking *in vivo* conditions• Possible high throughput• Control of environmental conditions• Imaging and analysis capabilities
• Disadvantages	• Costly and time consuming• Ethical concerns• Animal-human mismatch (size, loading, cells)• Poor control over the experimental conditions	• Largely based on 2D substrates• Low clinical predictivity• Lack of mechanical cues	• Rapid degradation of IVD structures• Poor control over the experimental conditions• Lack of mechanical cues	• Difficult to use• Low throughput• Bulky• Difficult to fabricate	• Not a clinically relevant scale• Challenging classic readouts (e.g. histological stainings)• Difficult to fabricate
	• Require dedicated animal facilities				

Despite large advances in macroscale *in vitro* and *ex vivo* IVD models, it is still challenging to use such systems to investigate fundamental questions on specific cell functions and molecular mechanisms involved in the conversion of mechanical loading to biochemical responses such as inflammation and pain. To improve the understanding of mechanotransduction and predict the success of new therapeutic approaches under loading, new *in vitro* models that enable both 1) recapitulation of the IVD native-like mechanically active environment at a cellular relevant scale and 2) compatibility with high-throughput setups, are warranted. Scalable, easy-to-use, and low-cost *in vitro* 3D loading systems would aid not only in fundamental research but also in advancement of disease-modifying and personalized therapies.

Organs-on-chip (OoC), also referred to as microphysiological systems (MPS), are emerging biomedical research tools derived from microfluidic technologies that allow a precise control over fluid behavior within micrometer-sized channels (Bhatia and Ingber, 2014; Esch et al., 2015). Owing to their three-dimensional scale, resembling the one experienced by cells in the body, cell-based OoCs can attain extraordinary control over cells behavior *in vitro* (Rothbauer et al., 2021). The main advantage of OoCs is their ability to mimic dynamic (even patient-specific) tissue microenvironments, while controlling crucial tissue parameters such as flow rates, molecular gradients, and biophysical cues (e.g. mechanical and electrical) (Zhang et al., 2018a; Ergir et al., 2018; Rothbauer et al., 2021). OoCs can achieve sufficient complexity to recapitulate traits of human pathophysiology, as already demonstrated for various tissues (Huh et al., 2010; Esch et al., 2015; Herland et al., 2020), thus potentially providing more predictable tissue responses than conventional *in vitro* models. Originally, OoCs platforms used the high control over the fluid motion and diffusion at the microscale to obtain finely tuned experimental conditions and mimic blood circulations in tissues including lung (Huh et al., 2010), gut, liver, and kidney (Herland et al., 2020). Microfluidic devices have also been extensively used to investigate the effects of shear stress in vasculoendothelial pathologies, both in 2D and 3D models (Chou et al., 2016; Kim et al., 2017). OoCs also represent cost-effective and compact 3D platforms that are compatible with parallelization and automation, thereby facilitating drug screening studies. They can serve as a tool for comprehensive evaluation of various cell types, biomaterials, drugs, and tissue-engineered products, potentially reducing the need for animal testing (following the Replacement, Reduction and Refinement (3Rs) principle). In certain cases, OoCs even demonstrated a higher relevance for predicting human responses, as animal models usually do not fully represent human conditions due to inter-species differences (Zhang et al., 2018a; Ergir et al., 2018; Rothbauer et al., 2021).

Recognizing the importance of mechanical factors in IVD homeostasis and degeneration, multiple loading-based macroscale *in vitro* models have been proposed and reviewed (Pfannkuche et al., 2020). Less attention has however been given to reviewing microscale OoCs aimed at modelling different aspects of the IVD pathophysiology. The main goal of this review is to explore the IVD-on-a-chip technology as a highly relevant tool to mimic and study human IVD pathophysiology in a medium-to high-throughput context. We focus on recent advances in OoCs, with applications in the IVD field together with devices that, while designed for other purposes, could benefit IVD studies (e.g. with the introduction of mechanical loading), and discuss their potential to evaluate new therapies. Finally, we discuss the limitations of the current approaches proposing the conceptualization of a prospective IVD-on-chip model that could be used in future mechanotransduction studies. This comprehensive review is intended for readers with different backgrounds ranging from medical and biological scientists to engineers.

## 2 Intervertebral Disc and Degenerative Disc Disease

**FIGURE 1:**
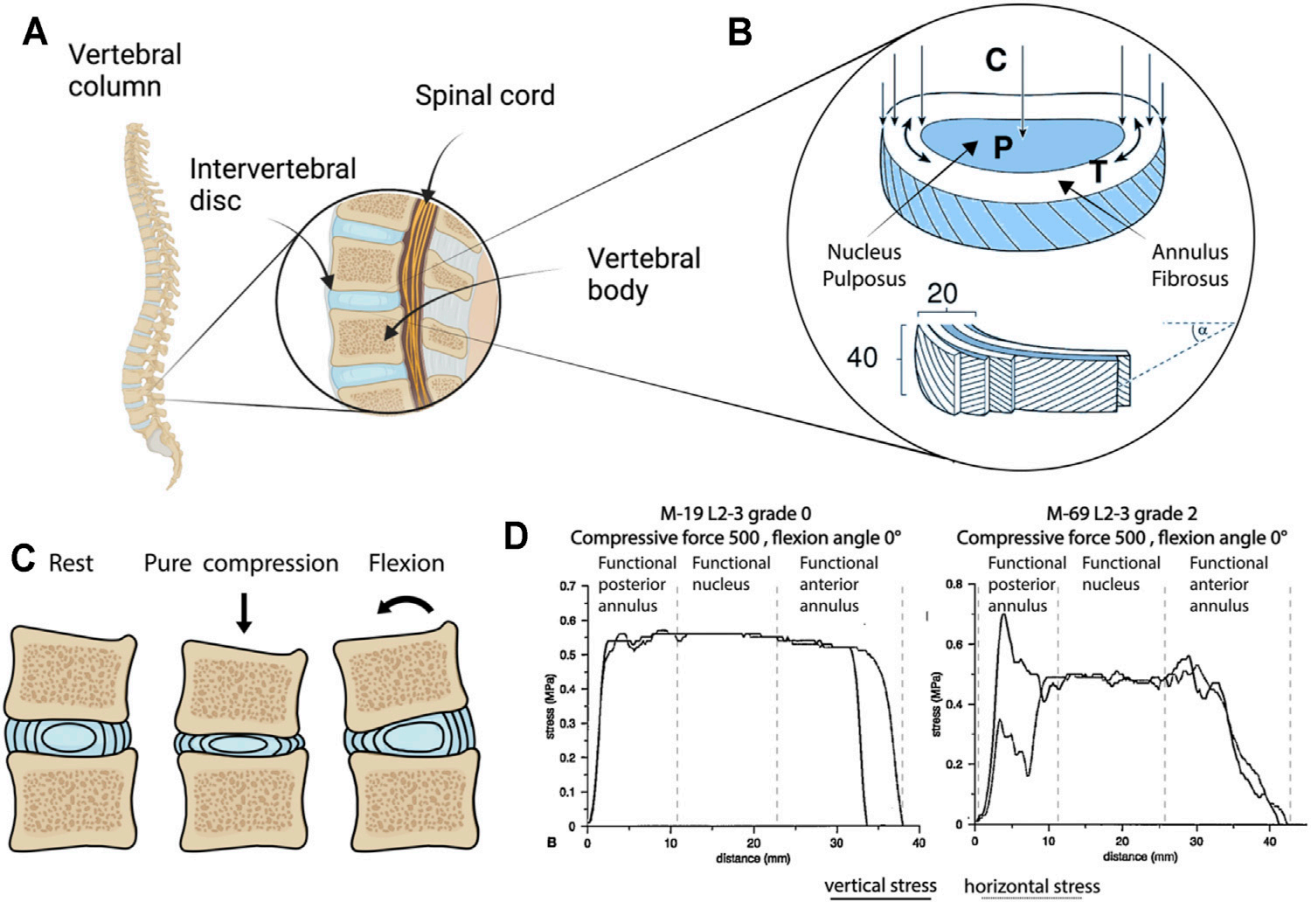
Intervertebral disc (IVD) anatomy and physical stimuli. **(A)** Vertebral column and the IVD (created with BioRender.com). **(B)** Structure, composition and main stresses acting on the components of the IVD. An overall vertical compression (C) of the IVD results in an increase of the hydrostatic pressure (P) in the nucleus pulposus (NP), which in turns tends to expand laterally causing an increment in the circumferential tension (T) experienced by the annulus fibrosus (AF), typically composed of 20 lamellae, each constituted by roughly 40 fibers disposed with a 30° angle (Adams and Roughley, 2006). **(C)** Schematization of the IVD in rest condition (i.e. when no stimuli are applied), following compression leading to a decrease in height and an outward expansion of the AF, and following a flexional slate (e.g. the one arising from someone bending their back). Bending in particular results in a complex stimulation state in the AF with the fibers on one side experiencing compression and on the other side experiencing tension. **(D)** Stress in human AF and in the NP of a grade 0 and a grade II (cadaveric IVD, as determined by stress profilometry) (McNally and Adams, 1992b; Adams, 2004). Images **(B)** adapted from Adams and Roughley (2006), **(D)** from McNally and Adams (1992b) and Adams (2004), reprinted with publisher permissions (SAGE Publications and Wolters Kluwer Health, Inc., respectively).

The IVD is a cartilaginous structure located between two adjacent vertebrae in the spinal column (Figure 1A). Anatomically, IVDs are constituted by a central nucleus pulposus (NP) encircled by the annulus fibrosus (AF) (Figure 1B), and connected to the neighboring vertebrae through hyaline cartilage endplates (CEPs) (Urban et al., 2004; Richardson et al., 2007). The NP is a gel-like structure predominantly composed of a loose network of highly hydrated proteoglycans (PGs) and collagen type II, with a PG/collagen ratio of 26:1 in healthy IVDs (Mwale et al., 2004). The AF is composed of circumferential lamellae (typically 15–25) formed by closely arranged fibers of collagen type I (Figure 1B). The external layer is composed of fibrous collagen type I fibers with a vertical direction connecting the cortical bone annular apophyses. Inner lamellae have fibers with a 30° orientation. Moving from the outside to the inside of the AF, the cellular population changes from fibroblast-like to chondrocyte-like cells responsible for the homeostasis of the fibrocartilaginous inner layers. CEPs, located above and below the IVD and separating the IVD from the vertebral endplates, are approximately 600-μm thick layers of hyaline cartilage rich in collagen type II and PGs. CEPs function as a mechanical barrier between the vertebral bodies and the NP but also as communication channels for nutrient transport from neighboring vascular channels into the IVD (Moon et al., 2013).

The IVD has the necessary mechanical properties to support the body weight and the flexibility to permit spinal movements. The lamellar structure of the AF provides load-bearing function, tensile resistance, and adequate support to maintain the NP pressure (Urban and Roberts, 2003), while the PG-rich composition of the NP mediates resistance to compression. A schematization of the forces to which NP and AF are subjected to, as originally depicted by Adams (2004), and Adams and Roughley (2006) is shown in Figure 1B. Compressive forces acting vertically on the IVD result in an increase of the pressure (P) in the NP and of the circumferential tension (T) in the AF.

During IVD degeneration, an imbalance between anabolic and catabolic processes occurs, leading to ECM degradation and functional changes. Degenerative disc disease (DDD) occurs when these changes are accompanied by chronic inflammation and pain. The first signs of DDD commonly manifest as reduced expression/accumulation of aggrecan and collagen type II in the NP, as well as an increase in pro-inflammatory cytokines (e.g. IL-1β, TNF-α) and ECM-degrading enzymes, namely matrix metalloproteinases (MMPs) and A Disintegrin And Metalloproteinase with ThromboSpondin motifs (ADAMTSs) (Wuertz and Haglund, 2013). The microenvironment of the degenerated NP is characterized by low levels of oxygen and glucose, acidic pH, high osmolarity, and complex non-physiological mechanical stress (Gantenbein et al., 2015; Vedicherla and Buckley, 2017), causing a catabolic shift (Johnson et al., 2015; Krupkova et al., 2016). Non-physiological loading and catabolism further reduce ECM turnover, leading to the development of microdamage, clefts, and tears in the AF (Urban et al., 2004). These changes are accompanied by the sensitization of sensory nerves by released nociceptive molecules and/or direct nerve damage, e.g. due to herniation or IVD space narrowing (Peng et al., 2005; Peng, 2013; Wuertz and Haglund, 2013). Furthermore, pro-inflammatory cytokines upregulate the expression of nerve growth factor (NGF), vascular endothelial growth factor (VEGF) and enhance the loss of PGs, which together allow for nerve and blood vessel ingrowth deeper into the IVD (Freemont et al., 2002; Binch et al., 2015). The ingrowth of newly formed nerves and vessels into the IVD was shown to aggravate LBP and to occur in patients with more severe symptoms (Freemont et al., 2001; Aoki et al., 2014a). IVD degeneration is also associated with CEPs becoming sclerotic, losing contact with the vertebral vasculature and exhibiting decreased permeability (Crock and Goldwasser, 1984). This process is considered to contribute to DDD progression by reducing the diffusion of nutrients to the cells of the NP (Ariga et al., 2001), but the correlation between CEPs and NP/AF degeneration was not completely clarified (Grignon et al., 2000).

Discogenic LBP is currently treated symptomatically by physiotherapy and pain medications. In a subset of individuals, these treatments fail and surgery is required. A surgery entails the risk of adverse effects, slow recovery, and high rates of reoccurrence (Henschke et al., 2008; Hoy et al., 2010). Notably, spine fusion (the surgical standard of care for DDD) fails to improve pain and quality of life in 20–30% of patients for various reasons (e.g. adjacent segment disease, implant instability) (Chou et al., 2002; Wei et al., 2013; Kenneth and Pettine, 2019). New approaches to treat DDD include disease-modifying molecular and cellular therapies (Vedicherla and Buckley, 2017; Smith et al., 2018). However, the therapeutic development is hindered by a poor understanding of mechanotransduction mechanisms in IVD degeneration, as well as by a lack of *in vitro* high-throughput drug testing platforms integrating relevant mechanical loading (Smith et al., 2018). In regenerative cell-based approaches, therapeutic cells are expected to support IVD regeneration by differentiating into IVD-like cells and/or by secreting trophic and anti-inflammatory factors to ultimately repair the IVD (Fontana et al., 2015; Sakai and Andersson, 2015; Wang et al., 2015; Meisel et al., 2019; Schol and Sakai, 2019). However, cells often fail to survive in the harsh IVD microenvironment and to adapt to the specific (often non-physiological) mechanical loading typically present in the degenerated IVD. There is therefore a high demand for developing new regenerative strategies compatible with (non-)physiological IVD mechanical loading and new models that recapitulate the altered mechanical environment *in vitro*.

## 3 Mechanobiology of the Intervertebral Disc

The IVD is commonly exposed to several types of mechanical loading including compression, tension, shear, torsion and their combinations. Spinal loading and PG content play major roles in the mechanobiological responses of the IVD. A schematization of the IVD loading status upon compression and flexion is reported in Figure 1C ***.*** The IVD experiences a diurnal change in intradiscal pressure according to the variation in day and night activity (Chan et al., 2011a). The majority of fluids in the IVD are absorbed by the negatively charged PGs in the NP that swell and provide compressive resistance. PG fixed charges are electrically balanced by cations in the interstitial fluid, mainly potassium and sodium. Upon application of a mechanical load, the NP loses water (but not ions), while the removal of the applied load causes rapid rehydration due to the osmotic gradient in the NP (Cramer and Darby, 1997; Urban and Roberts, 2003; Galbusera et al., 2014). The increased intercellular osmolality during loading draws water out of the cells, reducing the cell volume. Mechanical loading thus alters the physical environment of the IVD by causing changes in the water content and the chemical composition of the ECM and the cells (Chan et al., 2011a; Sadowska et al., 2018).

Mechanical stimuli elicit cellular responses in the IVD that depend on the magnitude, frequency, and duration of the loading (Sowa et al., 2011a). Spinal loading causes physiological dynamic compression of the IVD with a frequency between 0.2 and 1 Hz and diurnal variations in magnitude between 0.2 and 0.6 MPa (Chan et al., 2011b). Axial compression and swelling effects in the NP generate bulging and deformation of the AF, resulting in radial and circumferential tension with physiological strains up to 5.5% (Showalter et al., 2016). In general, a physiological level of mechanical loading is beneficial for IVD homeostasis, as it promotes solute transport and cell metabolism (Chan et al., 2011a). However, hyper-physiological mechanical stressors, caused for instance by impact, heavy weight lifting, altered muscle activations, and work/lifestyle factors (e.g. vibration exposure, gait, and posture) contribute to cell death, catabolism, and inflammation leading to IVD degeneration (Chan et al., 2011a; Fearing et al., 2018). Loading in the NP and in the AF changes also during DDD, as quantified in the early nineties by McNally and Adams (1992a) and reported in Figure 1D.

Most studies focusing on the role of mechanical loading of the IVD have investigated **c**ompressive stimuli. Static loading was shown to induce detrimental changes including downregulation of ECM genes, protease activation, and cell death both *in vitro* and *in vivo* (Ohshima et al., 1995; Iatridis et al., 1999; Lotz and Chin, 2000; Chen et al., 2004), supposedly via inhibition of nutrient transport and gas exchange (Fearing et al., 2018). Physiological dynamic loading (with a magnitude between 0.2 and 1 MPa) was reported to elicit anabolic responses with the promotion of cell metabolism and maintenance of ECM synthesis depending on magnitude and frequency (Maclean et al., 2004; Neidlinger-Wilke et al., 2005; Korecki et al., 2008). On the contrary, hyper-physiological dynamic compression at high magnitude (>1 MPa), low or high frequency (<0.1 Hz or >1 Hz), or long duration (>8 h per day) causes cell death (Walsh and Lotz, 2004; Wang et al., 2007; Illien-Jünger et al., 2010), decreased expression of anabolic genes as well as increased expression of MMPs, ADAMTS, and pro-inflammatory cytokines (MacLean et al., 2003; Ching et al., 2004; MacLean et al., 2005; Korecki et al., 2009).

Similar patterns were observed in tensile stress studies. Isolated AF cells stretched at low magnitude (1%) and physiological frequency (1 Hz) were shown to maintain proteoglycan production (Rannou et al., 2003). Another study found that low magnitudes (3 and 6%) and frequencies (0.1 and 0.5 Hz) of tensile strain downregulate catabolic mediators, while this effect is lost with higher magnitude (18%), frequency (1 Hz) and prolonged duration (24 versus 4 h) (Sowa et al., 2011b). Outside of the physiological window, stretching can even provoke detrimental biological responses. Cyclic stretching of IVD (mostly AF) cells at a high strain of 8–20% and at either hypo-physiological (0.001 and 0.01 Hz) or physiological frequencies (0.1–1 Hz) was shown to induce downregulation of anabolic markers (aggrecan, collagen II) (Wang et al., 2018) and upregulation of catabolic (MMP 1, 3, 9, 13, ADAMTS 4, 5) (Sowa et al., 2011a; Wang et al., 2018) and pro-inflammatory mediators (cyclooxygenase-2 (COX2), prostaglandin E_2_ (PGE2), interleukins (IL) 1β, 6, 8, 15, toll-like receptors (TLR) 2, 4, NGF, tumur necrosis factor alpha (TNF-α), monocyte chemoattractant protein (MCP) 1, 3, and monokine induced by gamma interferon (MIG)) (Miyamoto et al., 2006; Gawri et al., 2014; Pratsinis et al., 2016; Wang et al., 2018).

### 3.1 Mechanosensing and Mechanotransduction

Mechanosensing is defined as the process by which cells detect mechanical signals, while mechanotransduction is the process used by cells to convert mechanical signals into biochemical responses. While many studies have reported biological responses to mechanical signals in the IVD, the investigation and knowledge of the underlying mechanosensing and mechanotransduction mechanisms are limited. The response of cells to mechanical loading depends on cell morphology, cell-cell interactions, and cell-ECM interactions (Fearing et al., 2018). Mechanosensing occurs via surface receptors that activate intracellular mechanotransduction signaling pathways (Fearing et al., 2018).

Integrins are transmembrane heterodimers composed of α and β subunits that can bind specific ECM ligands depending on their subunits (Fearing et al., 2018). NP cells express the integrin subunits α1, α2, α3, α5, α6, αv, β1, β3, β5, β6, and β8, while AF cells express α1, α5, αv, β1, β3, β5, and β6 (Nettles et al., 2004). There is currently little information on mechanotransduction pathways through integrin binding. Interestingly, integrin-mediated mechanotransduction was shown to be altered in degenerated IVD cells compared to healthy controls (Le Maitre et al., 2009).

Integrins are part of focal adhesions, which connect the ECM to the cytoskeleton. In NP cells, F-actin is expressed as short dispersed filaments mainly at the periphery of the cell, while AF cells display organized fibers throughout the cytoplasm, especially in the outer AF (Fearing et al., 2018). Outer AF cells also express higher levels of β-actin compared to NP cells (Li et al., 2008). On the contrary, NP cells have a higher expression of tubulin compared to outer AF cells (Li et al., 2008; Fearing et al., 2018). Cytoskeletal reorganization is one of the mechanisms by which IVD cells respond to mechanical signals. It was shown that IVD cells stretched in silicone chambers realign perpendicular to the direction of stretching (Abbott et al., 2012) and express more actin filaments compared to controls (Li et al., 2011). Actin formation and organization are mediated by the RhoA/Rho-associated kinase (ROCK) signaling pathway (Amano et al., 2010). Interestingly, ROCK inhibition abolished cell-cell interactions and the formation of clusters in NP cells (Hwang et al., 2014). Cytoskeletal regulation of NP and AF cells has further been associated with the yes-associated protein (YAP) and transcriptional coactivator PDZ-binding motif (TAZ) signaling (Fearing et al., 2019; Wang et al., 2021). Cytoskeletal reorganization mediated by F-actin remodeling occurs also in response to hypo- and hyper-osmolarity resulting in cell volume changes (Fearing et al., 2018).

Mechanical stress or cell volume change caused by osmotic stress provokes conformational deformations of the cellular membrane that might open mechanosensitive ion channels (Liu and Montell, 2015; Fearing et al., 2018). Candidate ion channels that are expressed differentially between NP and AF cells were identified via proteomic analysis and included sodium, potassium, and calcium channels such as the recently investigated transient receptor potential (TRP) channels. TRP channels are non-selective calcium-permeable transmembrane channels that can be activated by different stimuli, including changes in temperature, pH, osmolarity, as well as oxidative and mechanical stress, either directly by mechanical forces applied to the cell membrane or indirectly via multistep signaling cascades that induce conformational changes, which in turn generate mechanical force on the cell membrane (Walter et al., 2016; Krupkova et al., 2017; Franco-Obregón et al., 2018; Kameda et al., 2019; Sadowska et al., 2019). The TRP vanilloid 4 (TRPV4) ion channel was recently identified as a mediator of stretch-induced inflammation and compression-induced cell damage and degeneration in IVD cells and tissues in the context of hyper-physiological mechanical loading (Cambria et al., 2020a; Cambria et al., 2021).

Another aspect to consider in IVD mechanobiology is its cross-talk with inflammation. During IVD degeneration, inflammation was shown to disrupt the F-actin network and osmotic stress-induced cell volume change in NP cells (Maidhof et al., 2014). Notably, inhibiting actomyosin contractility (with a myosin II inhibitor) mimicked the effects of inflammation on cell biomechanical properties, while increasing actomyosin contractility (using a RhoA activator) protected against the mechanobiological effects of inflammation in NP cells (Hernandez et al., 2020). Furthermore, actomyosin contractility was shown to regulate the nuclear translocation of the pro-inflammatory mediator nuclear factor kappa-light-chain-enhancer of activated B cells (NF-κB) in response to TNFα, controlling the downstream catabolic effects of this pathway (Hernandez et al., 2020). While gaps in IVD mechanotransduction knowledge still exist, these recent findings highlighted that protecting cell mechanobiological integrity is vital for the development of new approaches to prevent or reverse IVD degeneration (Hernandez et al., 2020).

## 4 Organs-on-Chip Mimicking the Intervertebral Disc

Understanding IVD’s functional anatomy, mechanobiology, and the changes occurring during DDD progression is instrumental in delineating the conditions to be replicated in OoC systems. The goal of OoCs is defined as “not to build a whole living organ but rather to synthetize minimal functional units that recapitulate tissue- and organ-level functions” (Bhatia and Ingber, 2014). The formulation of a clear experimental hypothesis is therefore paramount in adequately designing and exploiting this technology. Recently, the first attempts at developing microphysiological IVD models emerged in the OoC field. Here we present them grouping the proposed devices in 1) cytokine-based DDD models, 2) IVD-on-a-chip devices providing physical stimuli, and 3) devices that, initially designed to model mechanotransduction in other tissues, could be adopted for IVD studies.

### 4.1 Cytokine-Based IVD-on-a-Chip Models

**FIGURE 2:**
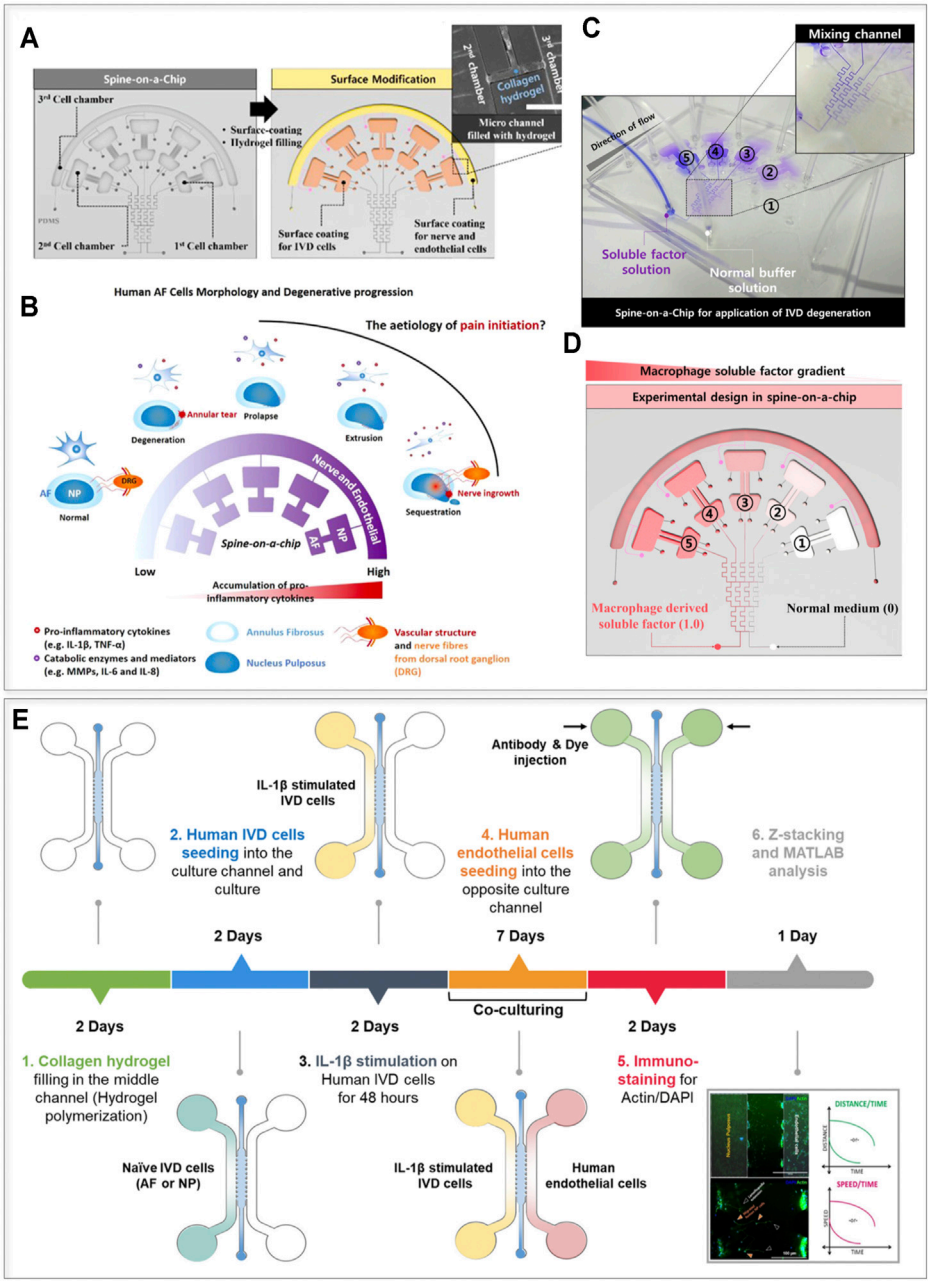
Cytokine-based IVD-on-a-chip models. **(A–D)** The layout and the possible conceptual adoption of the device presented by Hwang et al. (2017). **(A)** The device is composed of three chambers designed to culture AF, NP, and nerve or endothelial cells (Chamber 1, 2, and 3 respectively), and also comprises a gradient generator. Chambers 1 and 2, and 3 were coated respectively with fibronectin and with poly-D-lysine to facilitate cellular adhesion; the small channels connecting Chambers 2 and 3 were filled with a collagen-based hydrogel. **(B)** Conceptual use of the device. Different disease states are mimicked in the model as the cytokine concentration in the chambers increases (as indicated by the purple color). **(C)** Functional validation of the model. The gradient generator allows a concentration gradient in different culture chambers. **(D)** Design of an experiment investigating the effects of a gradient of macrophage-derived factors on IVD cells behavior. **(E)** The device and the experimental timeline of the study performed by Hwang et al. (2020). The authors used a simple device characterized by three channels separated by pillars. The central channel was filled with a collagen hydrogel while the lateral channels were filled with IVD (AF or NP) cells and human endothelial cells respectively. IVD cells were exposed to IL-1β and the effect of the stimulus on IVD cells and on the endothelial compartment in terms of cellular migration was studied. Images **(A–D)** adapted and reprinted from Hwang et al. (2017), **(E)** from Hwang et al. (2020), with publisher (API Publishing) permissions and based on http://creativecommons.org/licenses/by/4.0/, respectively.

While DDD-related pain may be caused by nerve root compression as a result of IVD protrusion, LBP also occurs in patients without nerve compression in MRI images (Loibl et al., 2019). This, not yet completely understood phenomenon, is possibly related to matrix alarmins and cytokine-mediated irritation of the dorsal root ganglia (DRG) pain channels and nerve endings located in the IVD (García-Cosamalón et al., 2010). Pro-inflammatory cytokines released by IVD cells and infiltrating macrophages promote the continuous breakdown of ECM components enabling the invasion of endothelial cells (ECs) and neurons into deeper IVD regions (García-Cosamalón et al., 2010). However, the effects of molecular gradients of inflammatory mediators, metabolic waste products or trophic factors on IVD pathophysiology are still not sufficiently explored. Hwang et al. (2017) developed a microfluidic device able to 1) generate gradients of pro-inflammatory and macrophage soluble factors, and 2) co-culture three different cell types, e.g. neurons, endothelial cells, and NP/AF cells. The device layout is reported in Figure 2A (Hwang et al., 2017). The conceptual functioning principle of the device and the achievement of molecular gradients are reported in Figures 2B,C, respectively. Exposure to either IL-1β (0–1 ng/ml) or to macrophage secreted factors (generated by THP-1 cells upon their exposure to phorbol myristate acetate) resulted in human AF cells upregulating the expression of inflammatory mediators (i.e. IL-6 and IL-8), degradative enzymes, and tissue inhibitors of metalloproteinases (TIMPs). Through the proposed platform it was possible to recapitulate the dose-dependent catabolic responses of primary human AF cells including morphological and kinetic cell alterations commonly found in the degenerated IVD. This model represents the first step towards a basic understanding of gradient-based mechanisms in inflamed IVD cells (Hwang et al., 2017).

It is apparent that numerous cross-talks exist between the IVD and adjacent structures, but how different cell types influence each other during IVD homeostasis and degeneration remains relatively unknown. Despite the potential of the device designed by Hwang et al. (2017) NP cells, neurons and vascular components were not actually used in this study. Neither the native (3D) architecture of the cells nor ECM components were introduced and the culture time was limited to 72 h. Provided such missing features are integrated, this model could also be adopted to elucidate basic pain mechanisms (e.g. through the NGF or VEGF gradients) (Freemont et al., 2002; Aoki et al., 2014b).

More recently, an IVD co-culture OoC was developed to investigate chemotactic invasion and migration of AF/NP cells and ECs in conditions simulating an inflamed IVD (Hwang et al., 2020). The device, schematized in Figure 2E together with the adopted experimental timeline, is composed of three distinct chambers separated by two rows of posts. The central chamber was filled with an (initially) acellular collagen hydrogel, while lateral chambers were seeded, respectively, with human, primary, naive or inflamed AF/NP cells, each cellular population in 2D on one side of the hydrogel. The authors demonstrated that AF/NP cells interact with ECs (immortalized human microvascular endothelial cells, HMEC-1) also suggesting a possible time line: AF cells respond to IL-1β early during the DDD course (resulting in an increased expression of IL6, -8, MMP1, 3 and VEGF family members), while NP cells interact with ECs by responding with higher levels than AF to ECs secreted factors (e.g. production of IL-6 and -8, VEGF, and MMP-3 was significantly higher in NP cells than in AF cells, under the presence of ECs conditioned medium). While the study gave a first insight into intracellular communications in IVD degeneration, paracrine signaling mechanisms involved in cell communication and migration were not characterized (Hwang et al., 2020). More advanced co-culture OoCs could reveal mechanisms modulating the recruitment of non-IVD cells (immune, endothelial, and neuronal cells) and enable precise targeting of pain mechanisms related to disease progression. Moreover, NP cells were still cultured in 2D contrarily to the physiological condition thus possibly disregarding the effect of ECM components (e.g. PGs) in determining cellular migration capacity and tissue chemical permeation. Notably, already existing OoC platforms could be exploited to culture AF/NP cells and also ECs in 3D to better investigate such mechanisms (Huang et al., 2009). Finally, it is worth mentioning that the adopted IL-1β concentrations (i.e. 10 ng/ml) were employed with the aim of obtaining a downstream effect rather than to reflect inflammation *in vivo* (Burke et al., 2002; Andrade et al., 2011; Altun, 2016).

### 4.2 IVD-on-a-Chip Models Providing Physical Stimuli

**FIGURE 3:**
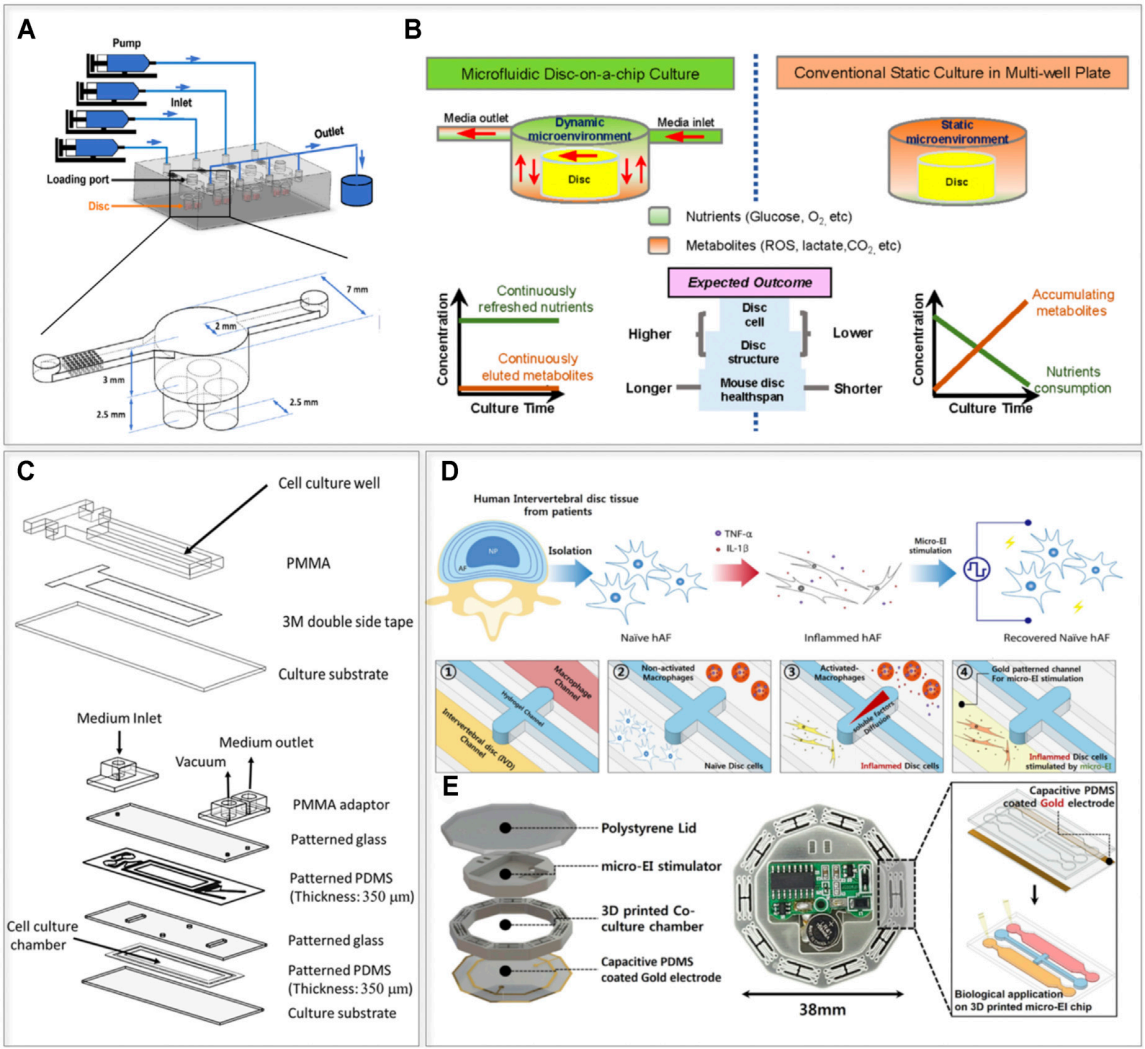
IVD devices providing physical stimuli. **(A,B)** Layout and the conceptual usage of the mouse disc-on-a-chip with controlled flow proposed by Dai et al., 2019). **(A)** The device is composed of four identical chambers, with three IVDs located in each chamber. A matrix of micropillars at the inlet of each chamber reduces the shear flow to which the whole mouse IVDs are exposed. **(B)** The system allows to keep constant levels of nutrients and metabolites resulting in the higher IVD culture times. **(C)** Layout of the shear stress device proposed by Chou et al. (2016). The top part shows the pre-culture chamber, while the bottom part shows the complete PDMS device. **(D,E)** The electrical stimulation device proposed by Shin et al. (2019). **(D)** Description of the experimental procedure from cellular extraction and stimulation to the compartments of the device. **(E)** Depiction of the different layers composing the electrically active device. Images **(A,B)** adapted from Xing et al. (2019) (Dai et al., 2019), **(C)** from Chou et al. (2016), **(D,E)** from Shin et al. (2019), reprinted with publisher (ACS Publications) permissions and based on http://creativecommons.org/licenses/by/4.

Incorporating controlled physical stimuli *in vitro* is essential to mimic human IVD pathophysiology. A key advantage of OoC, as compared to macroscale models, is the possibility of thoroughly controlling fluid flow conditions and parameters. The limited dimension of the channels assures a high laminarity of the fluid flow (the Reynolds number in microfluidic channels can be as low as 1), leading to a higher control of the phenomenon and an easier prediction of experimental conditions. The enhanced control over the fluid flow and consequently over the concentration of solutes and metabolites is also applicable in maintaining *ex vivo* explants, provided their dimensions are compatible with a microscale setup. On this regard, Dai et al. (2019) generated a microfluidic disc-on-a-chip characterized by continuous medium flow designed to accommodate whole mouse lumbar IVDs (8–10 weeks old). The introduction of perfusion chambers allowed an adequate flow exposure and fluid exchange, which improved cell viability and structural integrity in both NP and AF up to 21 days of culture, compared to static controls (Dai et al., 2019). A schematization of the device proposed by Dai et al. (2019) is reported in Figure 3A. Four perfusion units, each connected to a syringe (and a pump), were incorporated into the device. Each perfusion unit can host three IVDs thus increasing the experimental throughput. Notably, flow-induced shear stresses have been correlated to an altered response of IVDs in culture (Elfervig et al., 2001; Xia et al., 2015; Chou et al., 2016). Each perfusion unit was dimensioned to assure an adequate nutrient and metabolites exchange while minimizing shear stresses acting on the discs introducing a pressure dropping array of squared pillars at the inlet. Benefits of the constant chemical concentrations as reported by the authors (depicted in Figure 3B) include preserved cell viability, IVD structural integrity (i.e. maintenance of alignment and organization of AF lamellae and NP glycosaminoglycan (GAG) content), and conserved low expression levels of ADAMTS4, MMP13, TNF-α and IL-6, otherwise increased in static culture. While mouse IVDs might not be ideal for translational research and drug testing due to major structural and functional differences from human IVDs (Alini et al., 2008; Jin et al., 2018), they could be very useful to uncover the genetic basis of IVD degeneration and aging. As an example, using IVDs from excision repair cross complementation group 1 (ERCC1)-deficient mice provides the context of an aged tissue (Vo et al., 2013). This compact long-term microfluidic organ culture could consequently aid in advancing research on chronic IVD degeneration, mainly if dynamic loading was included in the model (Dai et al., 2019).

Many studies have shown that physiological compression and stretching promote IVD-like ECM formation. However, the effects of shear stress in the IVD are in general less explored. A preliminary assessment of the direct effect of shear stresses on human AF cells in a microfluidic setting was provided by Chou et al. (2016) (Figure 3C). The final platform was obtained through two culture passages. First, a polymethylmethacrylate (PMMA) pre-culture chamber (top part of Figure 3C) was connected to a fibronectin-coated substrate through double tape. After AF cells adhered to the substrate, this was detached from the culture chamber and assembled to the five-layer device (bottom part of Figure 3C). An inlet and an outlet were used for fluid flow while a vacuum inlet was used to apply a negative pressure to unite the culture substrate with the rest of the device. Shear stresses (1 and 10 dyne/cm^2^, ∼1 and 10 ml/h respectively) were generated in the cell culture chamber of the five-layer microfluidic device by a continuous flow of culture medium injected via a syringe pump. Shear stress distinctly influenced gene expression in AF cells, specifically collagen type I and MMP 1, in a value-dependent manner. The presented study was a first assessment of how AF cells respond to controlled shear. Cells were however seeded in 2D and stimulated with flow rates lower than those associated to physiological motions. Incorporating native 3D architecture could lead to substantial improvements in understanding molecular mechanisms of shear stress/interstitial fluid flow in the pathophysiology of IVD degeneration (Chou et al., 2016). Furthermore, AF cells from patients with IVD-related pathologies might show altered cellular responses, and therefore healthy controls or alternative cellular sources (e.g. derived from iPSCs) should be considered. On a technical note, the usage of vacuum to seal the device is acceptable for short experiments (e.g. a 4 h stimulation as used by the authors) but would not be sustainable for longer culture periods.

These two models provide a proof of concept of the potential of OoC platforms capable of physical stimulation for the study of IVD physiopathology. IVDs *in vivo* are however subjected to a complex strain field constituted by both compression (mainly in the NP) and stretching (mainly in the AF) of 3D structures. A better recapitulation of IVD physiology will therefore be necessary for the establishment of clinically relevant models.

Several OoC platforms include electrical sensors and/or stimulation systems (Maoz et al., 2017; Zhang et al., 2018b; van de Wijdeven et al., 2019). While IVD cells are not excitable by definition, different authors reported a modulatory effect of electrical stimulation (ES) on the expression of degradation markers and a beneficial ES effect on wound healing and inflammation responses (Zhao et al., 2006; Pavesi et al., 2016; Kim et al., 2009). A first attempt to include ES into an IVD microdevice, to recapitulate previously described effects of electrical stimulation on IVD cells (Miller et al., 2016; Wang et al., 2017; Kim et al., 2013), was made by Shin et al. (2019) (Figures 3D,E). The device hosted nine culture chambers. Each was constituted by two compartments where primary human AF cells and macrophages (i.e. TPA-activated THP-1 cells) were seeded in 2D and connected through a collagen I hydrogel filled compartment. A micro electrical impulse stimulator lodged in the middle of the device provided cells with ES at different frequencies (i.e. 100, 200, and 300 Hz) leading to beneficial effects on inflammatory mediators (TNF-α, IL-1β, IL-6, and IL-8) end ECM-degrading enzymes (e.g. MMP1). Similar to previously mentioned devices, this platform is limited by the brief culture time and the 2D configuration of AF cells. Such a microdevice could however be used in preliminary studies to investigate the therapeutic modulation of bioelectrical signaling (McCaughey et al., 2016), for instance in relation to the peripheral nervous system.

### 4.3 OoC Systems With Mechanical Loading Regimes Suitable for IVD Research

The discrepancy between *in vitro* cell monolayers and native 3D tissue structures results in many cases in altered phenotype, cell morphology and behavior. This renders results from 2D cell-based assays questionable, also in microfluidic based OoC platforms. By cultivating cells in hydrogels, scaffolds, or aggregates, it is possible to implement 3D cell culture systems that allow for an indirect mechanical stimulation. Varying the rigidity and stiffness of the ECM has been indeed shown to modulate cellular behaviors (Ergir et al., 2018). However, both IVD homeostasis and degeneration depend on the complex mechanical stimuli to which the spine is subjected, emphasizing the need for models that directly transmit key dynamic mechanical stimuli. While multiple macroscale bioreactors were designed to study the effect of mechanics, no report of mechanically active IVD OoC platforms is available. Recent developments in OoCs applied to other research fields (Rothbauer et al., 2021) could pave the way for IVD modelling towards more complex systems capable of a fine tuning of the biomechanical cellular stimuli. In this section we describe OoC mechanical devices that, although designed for different biological applications, could be adopted for the investigation of 3D NP and AF microconstructs under loading.

To date, only a few microdevices capable of recreating stretching and/or compression in 3D have been developed (Ergir et al., 2018). Marsano *et al.* (2016) proposed a miniaturized device designed to provide 3D murine and human cardiac cell constructs with controlled and tunable levels of mechanical strain (i.e. monoaxial stretching), which were initially applied to both healthy and pathological cardiac models (Marsano et al., 2016; Occhetta et al., 2018; Visone et al., 2021). A variation of the same device was recently applied in the cartilage field, showing how hyper-physiological confined compression of primary human articular chondrocytes cultured in 3D in a microdevice was sufficient to induce osteoarthritic traits (Occhetta et al., 2019). Specifically, the application of strain-controlled confined 30% compression triggered features of catabolism, inflammation and hypertrophy in the microtissue, similar to those found in clinical osteoarthritis (Occhetta et al., 2019).

**FIGURE 4:**
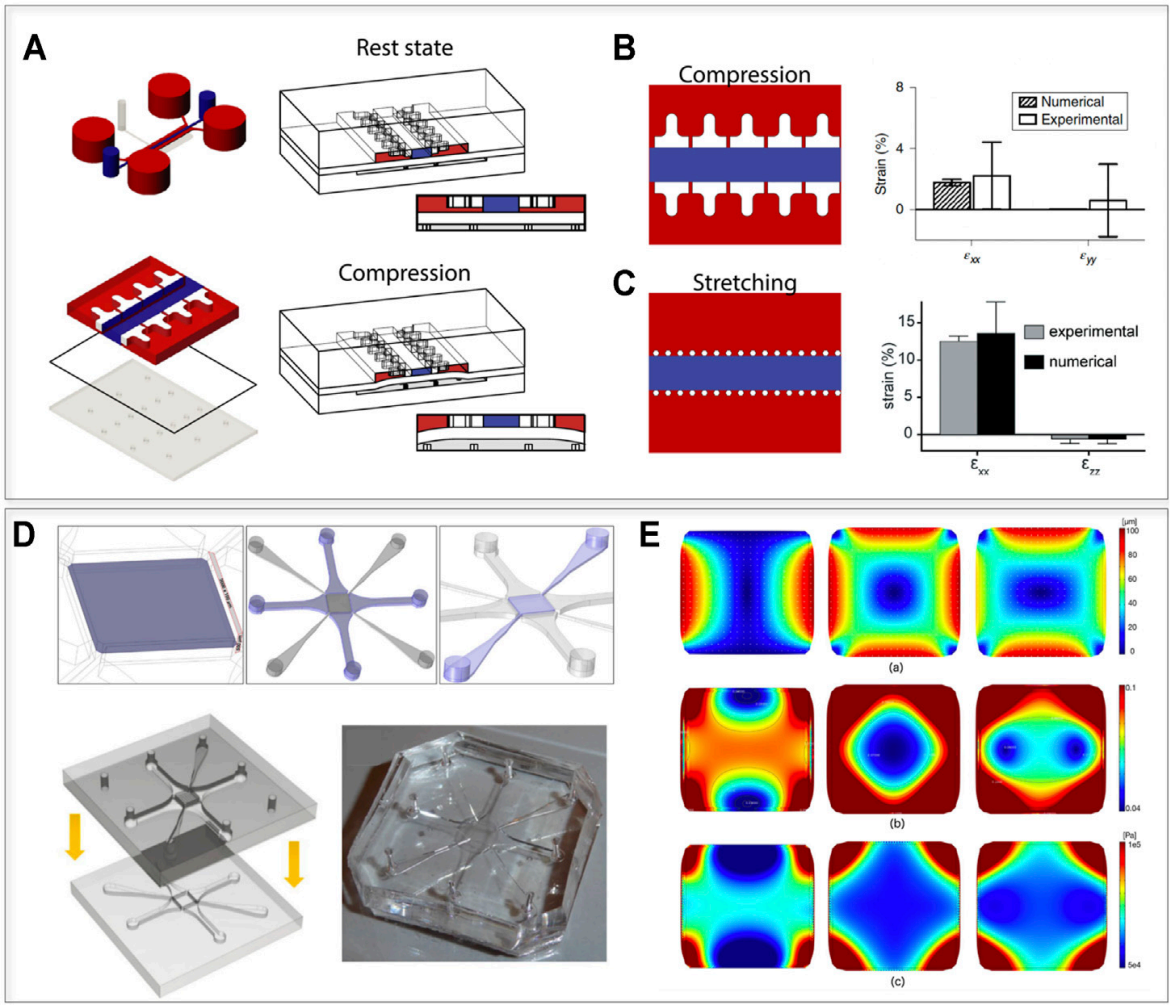
Possible technological transfer, introducing mechanics to IVD-on-chip models. **(A,B)** devices that allow subjection of 3D constructs to defined levels of confined compression or stretching, introduced by Occhetta et al. (2019) and Marsano et al. (2016). **(A)** Device layout and functioning principle. The device contains two chambers (a culture camber and an actuation chamber), divided by a flexible membrane. When a positive pressure is applied to the actuation chamber, the membrane bends upwards until it reaches the mechanical stop provided by two rows of overhanging pillars in the culture chamber. Regulating the distance between the pillars and the membrane, it is possible to apply a defined compression or stretching level. **(B)** Compression device and experimental evaluation of the lateral expansion of the device proposed by Occhetta et al. (2019). The T shaped pillars limit the lateral expansion upon compression resulting in an almost ideal confined compression state. **(C)** Stretching device and experimental evaluation of the lateral expansion of the device introduced by Marsano et al. (2016). Using hexagonal pillars with a wider spacing in between them upon compression the hydrogel in the central chamber expands laterally providing laden cells with a 10% stretching level. **(D,E)** The device layout and the displacement field of the device proposed by Gizzi et al. (2017) allowing complex displacements stimulation states of a porous membrane. **(D)** A central porous membrane is connected by four pneumatic chambers (that can be actuated electively to produce complex strain fields) and to four perfusion channels (highlighted in purple from left to right). The final device is obtained by bonding of two halves. **(E)** Evaluation of the strain field: (E.a) Displacement field induced on the porous membrane (PM) under uniaxial (left), equibiaxial (center) and biaxial 3:5 (right) loading patterns for a maximum pressure *p* = −500 mbar. (E.b) Color map and isolevel contours of the first invariant of deformation for the corresponding loading patterns. (E.c) Color map of the von Mises stress distribution for the three loading patterns. Images **(A–C)** adapted from Occhetta et al. (2019), **(D,E)** from Gizzi et al. (2017), reprinted with publisher (Springer Nature) permissions and based on http://creativecommons.org/licenses/by/4.

The same principle was recently incorporated in a multi-chamber mechanically active OoC device with an increased throughput (Mainardi et al., 2021). The device (Figure 4A) is composed of a top cell culture chamber and a compression chamber divided by a thin PDMS membrane. The culture chamber is constituted by a central hydrogel channel divided by two rows of overhanging pillars from lateral culture medium channels, with a gap between the bottom surface of the pillars and the flexible membrane. By applying a positive pressure to the actuation chamber, the membrane bends upwards compressing the 3D hydrogel. The compression level depends exclusively on the relative height of pillars and gap. The posts serve therefore the double function of 1) confining the 3D cell constructs and 2) defining a stroke length controlling a mechanical actuation mechanism. Tailoring the pillars geometrical section makes it possible to achieve a confined compression (e.g. through a T shape, Figure 4B) or monoaxial lateral stretching of the cellular construct (e.g. through widely spaced posts with a narrow hexagonal section, Figure 4C). Featuring both a 3D environment and mechanical stimulation, these devices could be used as a basis to study AF and NP cell responses to loading. Modulating the loading entity, it would be feasible to verify if these cellular populations respond differently to physiological and/or pathological strain levels.

Most of the mechanically active OoCs in literature (Ergir et al., 2018) focus on uniaxial loading while IVDs are subjected to complex strain fields due to spine flexion and torsion. Gizzi et al. (2017) introduced a microfluidic chip with multi-axial loading capabilities. The device is composed of a central porous membrane coupled with four vacuum chambers and perfused through perfusion channels. These features are evidenced in purple in Figure 4D, together with a schematization of the two layers used to close the device and a picture of a PDMS physical platform. While the authors did not report a specific biological application, they demonstrated how, by selectively applying vacuum to the chambers, the device could subject the porous membrane to specific strain fields (i.e. uniaxial, equibiaxial, and biaxial strains). This could be useful in the replication of the complex deformations to which AF fibers are subjected *in vivo*. The displacement field, the strain map, and the von Mises stress obtained on the porous membrane for uniaxial loading, equibiaxial loading and biaxial loading are reported in Figure 4E ***.*** Notably, the authors adopted a computationally informed design procedure to optimize the achievable strain field. A similar approach could be introduced to replicate the stimulation patterns characterizing IVDs. The main developmental steps towards such mechanically loaded IVD OoCs should thus include device design and *in silico* mechanical characterization, validation of cellular mechanotransduction mechanisms, and subsequent recapitulation of physiological and non-physiological IVD phenotypes upon loading.

## 5 Perspective on Design Criteria for IVD-on-a-Chip

In the last decade, OoCs have been proven able to recapitulate relevant organs and tissues functionalities *in vitro* by providing native-like biochemical and biophysical cues to 3D cell (co)-culture within biomimetic microarchitectures. Among others, the interest around mechanically active OoC is growing with their ability to recapitulate mechanical stimuli that modulate physicochemical cell and tissue responses. OoCs are an ideal tool to capture mechanobiological interactions, with the capacity of 1) providing a fine control over spatiotemporal organization of *in vivo*-like tissue architecture, 2) precisely controlling the magnitude, duration, and frequency of the biomechanical stimuli, and 3) monitoring in real time the effects of applied mechanical forces on cell, tissue, and organ functions [84]. In order to investigate load-associated mechanisms in the human IVD, an ideal microscale device should arguably be capable of recapitulating the IVD anatomy/physiology and, at the same time, allow precise *in situ* analysis of at least the two major IVD cell types (NP and AF) experiencing specific physiological loading stimuli in 3D (e.g. compression and stretching). Here we discuss the features and the parameters that should be considered in a foreseeable IVD-on-a-chip which could be useful in IVD pathophysiology investigations.

It is necessary to note that OoCs are almost exclusively strain regulated (i.e. the parameter set by the user is the strain amount) and not load based, in contrast to macroscale bioreactors. Physiological variations and frequency can easily be obtained at the microscale but measured variations in IVD pressure (0.2–0.6 MPa) (Chan et al., 2011a; Chan et al., 2013; Rosenzweig et al., 2016) are difficult to achieve since actuation pressures in OoC are in the order of 10^−2^ MPa. Moreover, forces acting on the IVDs are orders of magnitude higher than those that can be replicated at the microscale and with the adoption of soft hydrogels as a substrate. It should therefore be kept in mind that while a stricter control on the mechanical stimuli is achievable, it is paramount to properly translate a macroscale stimulus into a microscale one. The ideal IVD-on-a-chip might therefore depend on the specific downward effect to be replicated or the parameter to be evaluated. In patient-oriented research, an ideal IVD-on-a-chip could be designed aiming at replicating all conditions that characterize a DDD (e.g. altered compression levels, inflammation, low pH) to determine if a given therapeutic option, like the supplementation of cells with restorative capacities (Gryadunova et al., 2021), could withstand the cited conditions.

### 5.1 Device Concept

The device could be constructed taking inspiration from the described works of Hwang et al. (2020). Featuring compartments for the co-culture of different cellular populations, but adapting the designs that allow the 3D culture of different cellular populations (i.e. NP cells in the middle and AF cells in lateral compartments), together with channels for medium supplementation (Huang et al., 2009). With the use of pillars positioned in between the compartments and/or different actuation chambers, it could also be feasible to apply controlled mechanical stimuli differentiating between the NP (subjected to confined compression) and AF (subjected also to strain) and/or to apply complex stimuli (e.g. reminiscent of those experienced by IVDs during flexion). Effective device structures, layout, and dimensions will require a careful design procedure that factors in 1) the stimuli of the different compartments *in vivo*, 2) the necessity of an adequate supply of nutrients, but also the possibility of subjecting the compartments to DDD chemical stimuli and 3) an adequate tradeoff between complexity and usability.

The catabolic microenvironment in the degenerated IVD negatively influences cell survival and function, with mechanical loading being a possible aggravating (or conciliating) factor. While the AF and NP are the most evidently affected compartments, DDD also involves the recruiting of endothelial cells and nerves from the DRGs (Hwang et al., 2017). In an ideal IVD OoC, different cell types would be co-cultured in separate chambers connected directly or through microchannels to allow for paracrine signaling and diffusion of compounds. However, including compartments for other cells in the same device might further increase the operational complexity (and introduce possible confounding cross-talks). Herland et al. (2020) recently proposed a methodology to couple different OoC models in an effective and automated way. Cross cellular signaling between IVD cells and other compartments could therefore be achieved by fluidically coupling different devices.

Another aspect concerning the device layout regards the incorporation of on-chip biosensing capabilities (recently reviewed in (Ferrari et al., 2020)). Over the last years, a range of biosensing approaches embedded within microfabricated OoC systems have been reported including biosensors for monitoring cell growth and behavior, electrical and mechanical properties, and environmental parameters such as oxygen, pH, and metabolites. The integration of biosensors enables a continuous and non-invasive monitoring of microtissue evolution and dynamic measurements of cellular responses to diverse stimuli, thus providing detailed information about microtissue behavior at the molecular level (Ferrari et al., 2020). An integrated approach coupling a mechanical actuation compartment and biosensors on-chip would thus not only allow a precise control of the magnitude, duration, and frequency of the biomechanical stimuli but also the real-time monitoring of their effects on cell functions (Ergir et al., 2018). These advanced microphysiological IVD models could therefore be used to gain an in-depth understanding of the molecular mechanisms underlying load-induced IVD homeostasis and degeneration, and the role of mechanotransduction in regenerative feedback loops.

### 5.2 Biomaterials

Embedding cells in 3D OoC platforms often requires the injection of a cell-laden formulation while it is polymerizing. This procedure leads to the necessity of adopting hydrogels instead of solid scaffolds and demands an accurate evaluation of the gel usability (e.g. ease of handling, polymerization rate, speed) and biological relevance (e.g. analogy with the studied tissue ECM). The structure and composition of a biomaterial are critical for cell mechanosensitivity, as they influence how cells react to applied loads. For example, it is known that the cellular responses to substrate stiffness differ between AF and NP cells. AF cells seeded on a stiff substrate (in 2D) display an elongated morphology and distinct actin fibers, while they are round with less clear actin fibers on soft substrates [96]. NP cells tend to form clusters on soft substrates with cell-substrate and cell-cell interactions mediated by cadherins [96]. Concerning the IVD, hydrogels with non-physiological ECM porosity or non-fibrous matrices are not representative of the cell environment in the real tissue but they might be optimal in terms of handling and homogeneity. A possible circumvention of the obstacle could be the adoption of biodegradable materials that although not initially IVD-like, allow IVD cells to generate their own ECM as they are gradually degraded. This requires a preculture period for the achievement of a healthy IVD model before mechanical loading is applied and its effects evaluated (Occhetta et al., 2019). Hybrid materials combining the stability of synthetic matrixes with the binding motifs necessary for cell-matrix interactions and mechanotransduction signaling activation were already introduced (Ehrbar et al., 2007). Recently, an agarose-collagen hydrogel has been developed to mimic both the non-fibrillar (i.e. PGs) and fibrillar (i.e. collagen fibers) components of the IVD matrix (Cambria et al., 2020b). This composite biomaterial is suited for mechanotransduction studies as it combines the mechanical strength of agarose with the biofunctionality of collagen type I and could be a candidate for the study of loading effects in microphysiological settings.

### 5.3 Cellular Sources

Most of the cited IVD-related studies adopted either NP or AF cells from patients undergoing elective IVD surgeries. While the effect of different stimuli (e.g. shear stress or cytokine administration) could be determined in these studies, a more complete assessment of the mechanisms leading to DDD would require the application of a defined stimulus to healthy cells. However, the procurement of healthy IVD cells is limited by the low availability of suitable donors, yield, and proliferation of human primary IVD cells. Moreover, the presence of different cells types and spatiotemporal variations of IVD cell phenotype further complicate our understanding of IVD biology (Pattappa et al., 2012). Young human NP (but not adult NP) were shown to contain notochordal cells, while adult NP, AF, and CEPs contain tissue-specific progenitors with enhanced regenerative properties (e.g. multipotent NP progenitor cells (NPPC) in the NP) (Pattappa et al., 2012; Sakai et al., 2012; Tekari et al., 2016). However, investigation of these cell types has been challenging due to their very low yield and the fact that their numbers might further decrease with age and degeneration (Sakai et al., 2012; Tekari et al., 2016). The scale reduction towards OoCs leads to a consistent decrease in the number of required cells with respect to classic 2D models and to macroscale bioreactors, increasing the number of experiments/conclusions that can be generated from a single healthy IVD biopsy (e.g from cadaveric donors) but also from other often limitedly available cellular sources. These include transfected or CRISPR-edited populations, whose number is reduced by gene editing itself and antibiotic selection (Krupkova et al., 2018). With their ability to differentiate into NP-like cells, induced pluripotent stem cells (iPSCs) could also be considered as a valid alternative adult cell source (Tang et al., 2018). A loaded IVD microtissue generated using cells derived from autologous iPSCs could possibly be employed in research on patient-specific DDD mechanisms and personalized drug development. IVD studies performed so far largely disregard the involvement of CEP chondrocytes in the DDD pathogenesis. The incorporation of CEP chondrocytes in OoC IVD models could give new insights into their crosstalks with NP/AF cells. Co-culture OoC IVD models could aid in revealing interactions between different cell types, uncover mechanisms responsible for regenerative functions of tissue-specific progenitors, and/or investigate strategies to enhance load-induced paracrine functions of specific IVD cell types.

## 6 Conclusion

OoCs are *in vitro* microscale models that by recapitulating the cell-cell and cell-ECM functional architecture, the tissue-tissue interfaces, and the physicochemical environment of human tissues and organs, produce levels of analogy not achievable with classic *in vitro* cultures (Bhatia and Ingber, 2014). These functionalities are made possible by the versatility and the ease of prototyping of microfabrication techniques that allow the introduction of gradient generators, multiple culture chambers, mechanical actuators, and biochemical sensors within coin sized devices. In the context of IVD in general or the DDD pathology specifically, OoC systems could thus be configured to study aspects of the disease such as the effect of different mechanical stimulation levels and the exposure to various concentrations of pro/anti-inflammatory factors.

In this review we briefly delineated the IVD anatomy and the mechanosensing and mechanotransduction mechanisms responsible for pathophysiological IVD responses, and described how different microscale *in vitro* models were used as instruments for IVD/DDD research. In particular, we reported devices that 1) permit the co-culture of different cells involved in the DDD pathogenesis (i.e. NP cells, AF cells, neurons and endothelial cells); 2) allow the long-term preservation of mice IVDs; and 3) expose IVD cells to physical stimuli such as shear stresses or electrical impulses. While various OoC models are available for other musculoskeletal tissues (for instance bone-on-chip models as reviewed by Mansoorifar et al. (2021)), the application of this technology to the IVD field is still relatively budding and progress will be needed to develop representative IVD models. New mechanically active OoCs can be designed, or existing OoCs developed for other purposes could be repurposed for the replication of IVD loading. Mechanically active OoCs will allow the determination of stimuli that elicit degeneration in specific cell types, and if a given compound (e.g. mechanotransduction inhibitors such as TRPV4 agonist (Cambria et al., 2020a)) can reduce this phenotype while the injurious mechanical stimulus persists.

Increasing evidence highlights the involvement of other spine components in DDD symptomatology, e.g. detrimental age-related biological changes in CEPs (Fields et al., 2018). A full recapitulation of the architecture of all three tissues is challenging at the microscale but fluidically connected chambers could be adopted, for instance to determine how CEP chondrocytes exposed to altered mechanical stimuli effect NP and AF cell phenotype. Similarly, recent advances involving the incorporation of neuronal cell components and gradients of pain signals into OoC devices could enable progress towards analyzing discogenic pain-on-a-chip.

Little is known about *in situ* interactions between therapeutic and resident IVD cells under loading. Mechanically simulated OoCs could facilitate the optimization of strategies to increase adaptation and integration of cell-based grafts upon implantation (Huang et al., 2014; Sakai and Grad, 2015) in an easily controllable environment. Numerous IVD studies made use of mechanically active macroscale bioreactors, which are more suitable to accommodate whole IVDs, where specific injury characteristics (e.g. annular tears) can be recapitulated *ex vivo*. The concept of OoC does not intent to recapitulate whole IVDs but rather exploits the high experimental parameters control for mechanistic cause-effect investigations.

Overall, numerous further advances are required to achieve a representative IVD-on-a-chip. With the aim of augmenting our understanding of the mechanisms that lead to painful IVD degeneration, OoCs allow a higher throughput and require minimal (in the range of microliters) volumes of solutes and cell numbers while still retaining the capacity to generate 3D microtissues with chemical and mechanical stimuli, and even integrated biosensors.
